# Identification of allergens in coconut milk and oil with patients sensitized to coconut milk in Sri Lanka

**DOI:** 10.1186/s12948-022-00181-0

**Published:** 2022-12-20

**Authors:** Janitha Iddagoda, Peshala Gunasekara, Shiroma Handunnetti, Chandima Jeewandara, Chandima Karunatilake, Gathsaurie Neelika Malavige, Rajiva de Silva, Dhanushka Dasanayake

**Affiliations:** 1 Institute of Biochemistry, Molecular Biology and Biotechnology, University of Colombo, Colombo, Sri Lanka; 2 Department of Immunology, Medical Research Institute, Colombo, Sri Lanka; 3 Allergy, Immunology and Cell Biology Unit, Department of Immunology and Molecular Medicine, University of Sri Jayewardenepura, Nugegoda, Sri Lanka; 4 Teaching Hospital, Peradeniya, Kandy, Sri Lanka

**Keywords:** Allergens, Anaphylaxis, Coconut milk, Coconut oil, IgE reactivity

## Abstract

**Background:**

Despite the low prevalence of IgE sensitivity to fresh or boiled coconut milk and coconut oil, those may contain allergens of which the clinical significance remains undetermined. This study aimed to identify and compare allergens in fresh coconut milk (FCM), boiled coconut milk (BCM), unrefined wet-processed coconut oil (WPCO), and dry-processed coconut oil (DPCO) using sera from patients with allergy to coconut milk.

**Methods:**

The study included 18 patients with immediate hypersensitivity to coconut milk, including five who developed anaphylaxis. Sensitization was assessed by skin prick test and ImmunoCAPs using commercially available coconut extracts. Immunoblotting was performed to identify and compare allergen profiles.

**Results:**

Total sIgE levels and overall IgE reactivity of patients with anaphylaxis were higher compared to patients with allergy. Twelve allergens ranging from 5 to 128 kDa including six novel allergens with 5, 12, 47, 87, 110, and 128 kDa were visualized in immunoblots with FCM. Similarly, nine allergens of 5, 12, 17, 32, 35, 47, 87, 110, and 128 kDa were detected in BCM. One allergen (110 kDa) was discerned in all four extracts. Higher IgE prevalence was detected with three allergens of 55, 87, and 110 kDa.

**Conclusions:**

Allergens of BCM and unrefined coconut oil (WPCO and DPCO) were determined for the first time. Novel allergens of 87 and 110 kDa and the 55 kDa allergen have the highest potential to be used in Component Resolved Diagnostics. Further, these findings demonstrate that, patients who have an allergy to coconut milk could also react to boiled coconut milk and unrefined coconut oil.

## Introduction

Allergy to Coconut (*Cocos nucifera*) is not rare in Sri Lanka though it is a popular food among Asians [1]. Coconut, a drupe of order-Arecale and family-Arecaceae, contains fleshy meat inside amid testa, endocarp, mesocarp, and exocarp. Fresh coconut and milk or oil, extracted from the meat (kernel) is a major part of Sri Lankan and Asian cuisine.

Previous studies have reported allergic reactions following the consumption of coconut; milk, cream, oil, and water containing food. A recent Australian pediatric case series has reported 35 patients with type 1 hypersensitivity to coconut including 9 patients with anaphylaxis to coconut milk, coconut cream, baked coconut, and coconut water [2]. Another allergy study from the United States has reported 69 patients with allergic reactions to coconut, including 2 during breastfeeding, 10 after contact, and 57 following ingestion, with 50% of the patients who developed allergy after ingestion having mild/moderate anaphylaxis [3]. In addition, several other studies have reported allergy/ anaphylaxis following ingestion of fresh coconut, coconut milk, and other coconut containing food with the total of 10 cases reported across all studies [4–13]. Allergy to coconut oil is rare and only two cases have been reported [5, 14]. Further, several other cases of contact dermatitis have been reported as a result of sensitization to coconut oil derived surfactants; cocamide DEA (diethanolamine), cocamidopropyl betane, and TEA-PGE-3 (triethanolamine-phenyl glycidyl ether-3) cocamide sulphate [15–23]. Coconut pollen is a major inhalant allergen in the Indian subcontinent [24]. A case of occupational allergic conjunctivitis in a patient caused by coconut dust has been reported as well [25].

Previous molecular studies have identified a set of allergens; 16, 18, 20, 22, 25, 27, 28, 29, 30, 32, 35, 36.5, 39, 50, 55, 66, 75, 78, and 80 kDa in fresh coconut and milk [6–13]. Of these, the 29 kDa allergen is a 7S globulin (Coc n 2), whereas the 35 kDa allergen is a subunit of cocosin, a 11 S globulin (Coc n 4) [7, 8, 26]. 7S globulins and 11S globulins are also known as vicilin-like proteins and legumin-like proteins respectively. These are seed storage proteins categorized under the Cupin superfamily. Coc n 1, the only coconut allergen included in the official allergen nomenclature subcommittee of the International Union of Immunological Societies (IUIS), is a vicilin-like protein of 53 kDa identified as a novel allergen in coconut pollen [24, 27]. The same study reported 11 more inhalant allergenic proteins, including 11S globulin, enolase, and isoflavone in coconut pollens [24]. None of the studies have determined the allergenicity of boiled/cooked coconut milk or coconut oil.

Coconut oil can be broadly categorized into wet- processed coconut oil (WPCO) and dry-processed coconut oil (DPCO) [28, 29]. DPCO, which is the most popular form, is mostly refined and extracted from older, dried coconut kernels called copra [28]. Dry processed, unrefined coconut oil (crude coconut oil) is also edible and widely used. WPCO or virgin coconut oil is mostly unrefined and extracted from fresh coconut meat [28]. The protein content of coconut oil depends on its extraction and refining process. Generally, refined coconut oil is pure and free from protein contaminants [28]. Unrefined oil contains nearly 30 times more proteins (250 µg/ml) compared to refined coconut oil (7.9 µg/ml) [30]. Unrefined soybean oil has a residual allergenic potency, which may similarly apply to unrefined coconut oil as well [31]. However, the clinical significance of this possibility is yet to be determined. Most of the patients who are allergic to coconut milk can consume coconut oil. However, there is one case report where the patient was allergic to both coconut milk and coconut oil [5].

Coconut is distantly related to tree nuts and legumes even though some in vitro studies have shown IgE cross-reactivity with hazelnut, walnut and 7S and 11S globulins of lentils and soybean [8, 10, 13, 32, 33]. Co-sensitization between coconut, tree nuts and legumes has been described using sIgE levels [3, 32]. Cross-reactivity between coconut and latex has been clinically proven [34].

The current diagnosis of coconut allergy relies on skin prick testing and in vitro Phadia ImmunoCAP (f36) testing. There are no commercial component resolved diagnostic (CRD) reagents available for coconut allergy. Cross-reactive Carbohydrate Determinants (CCDs) are frequently found in plant-derived food and give rise to anti-CCD IgE which are clinically irrelevant. Hence, CRD with recombinant ImmunoCAPs of coconut specific allergenic proteins would be more productive in obtaining accurate results without any effect of anti-CCD IgE.

This study included 18 Sri Lankan patients with immediate hypersensitivity to fresh or boiled coconut milk, including 5 who had anaphylaxis. This cohort also included a patient with allergy to both coconut milk and coconut oil. It is clinically important to determine the allergenicity of fresh coconut milk (FCM), boiled coconut milk (BCM), and also coconut oil in order to educate patients regarding which form of coconut they should avoid or can consume. Therefore, the aim of this study was to evaluate the clinical features of coconut allergy, and to identify allergens and shared allergens in FCM, BCM, and unrefined WPCO and DPCO which can be useful in future diagnostic and therapeutic strategies.

## Methods

### Ethics clearance

Ethics clearance was obtained from Ethics Review Committee, Medical Research Institute, Colombo, Sri Lanka (ERC no: 11/2019).

### Patients and controls

Patients (n = 18) who developed allergy, including anaphylaxis (n = 5), following ingestion of coconut milk and/or application of coconut oil were recruited. Clinical data were obtained using an interviewer administered questionnaire. Sensitization to coconut milk (fresh) was confirmed using skin prick testing. In addition, medical records were reviewed to gather additional information (Table 1). Blood samples (5 ml) were collected from patients and healthy controls (n = 5) after obtaining informed written consent. Serum was separated from each sample and stored at − 20 °C.

**Table 1:** Details of patients with coconut milk allergy and their specific IgE levels

Patient No	Age (years)	Gender (Male/Female)	Skin prick test (±)	Specific IgE (f36) kUA/L^**†**^	Symptoms with	Other allergies
1	13	Female	+	13.20	Anaphylaxis (Urticaria, Angioedema, Vomiting, Difficulty in breathing)	Pea, Chickpea
2	1 ½	Female	+	35.40	Anaphylaxis (Urticaria, Angioedema, Difficulty in breathing)	–
3	6	Female	+	13.90	Anaphylaxis (Urticaria, Angioedema, Vomiting, Difficulty in breathing)	–
4	2 ½	Female	+	20.90	Anaphylaxis (Urticaria, Angioedema, Vomiting, Difficulty in breathing, Cardiac arrest, Loss of consciousness)	–
5	2/3	Female	+	4.22	Anaphylaxis (Urticaria, Angioedema, Vomiting, Difficulty in breathing)	Cashew
6	32	Female	+	1.39	Urticaria, Angioedema	Aeroallergens, Egg, Lentils
7	2 ½	Female	+	8.70	Angioedema	–
8	1 ½	Male	+	2.40	Urticaria, Angioedema	Cow's milk
9	2	Male	+	4.35	Urticaria, Angioedema	–
10	5	Male	+	1.31	Urticaria	–
11	1 ½	Female	+	8.34	Urticaria, Angioedema	Cow's milk, Lentils, House dust mite
12	3	Male	+	4.35	Urticaria	–
13	1	Male	+	2.36	Urticaria, Angioedema	–
14	3/4	Male	+	51.30	Urticaria	Cow’s milk
15	4	Male	+	17.10	Urticaria, Cough, Hoarseness	–
16	2/3	Female	+	0.06	Angioedema	–
17	2/3	Female	+	0.44	Urticaria, Angioedema	–
18^±^	2	Female	+	0.95	Urticaria and Angioedema to coconut milk and oil, Redness of eyes to coconut oil fumes	Lentils, Soybean

^±^Allergic to coconut oil as well

^**†**^Phadia immunoCAP for coconut. Total sIgE levels were significantly higher in patients with anaphylaxis compared to patients without anaphylaxis (P = 0.04)

### Sample preparation

Coconut milk was extracted from fresh scraped coconut kernel by pressing. A portion of extracted FCM was boiled up to 70 °C for 15 min to determine its allergenicity after cooking/boiling. WPCO (unrefined) was extracted by condensing coconut milk overnight at 4 °C followed by prolonged boiling. DPCO (unrefined) was extracted by pressing copra using a copra oil expeller followed by sedimentation and filtering.

### Protein extraction and quantification

Proteins of unrefined WPCO and DPCO were extracted according to the Acetone: Hexane (AH) extraction method described by Martín-Hernández, Bénet, and Obert, 2008 [35]. FCM and BCM were directly used for allergen identification without extracting proteins. Proteins of each sample were quantified by the Bradford assay [36].

### Sodium dodecyl sulfate polyacrylamide gel electrophoresis (SDS-PAGE)

Proteins of FCM, BCM, WPCO, and DPCO were separated according to their molecular weights by SDS-PAGE in 12% polyacrylamide gels under reducing conditions using a mini protein R-apparatus (Bio-Rad).

Samples were prepared prior to electrophoresis. Both fresh and boiled coconut milk were diluted 15 times with 0.13 M PBS (pH- 7.2). 15 µl of diluted coconut milk (fresh and boiled) were mixed with 5 µl of 4X Laemmli sample buffer. 20 µl of proteins extracted from wet-processed and dry-processed coconut oil were mixed with 7 µl of 4X Laemmli sample buffer. The samples were denatured at 95 °C for 5 min before loading into the gel. Loaded samples and 6 µl of protein standards (7.1 to 209 kD) were electrophoresed at 70 V at 4 °C for 2 h.

Thereafter, the gel was stained using Coomassie Brilliant Blue R-250 solution for 2 h, followed by de-staining for 4 h. Values of retention factors (Rf) of the protein bands were calculated and molecular weights of the proteins were estimated using a standard curve of pre-stained protein markers.

### Immunoblotting

Immunoblotting, as described in our previous reports [37, 38], was carried out to determine the presence of specific IgE to fresh coconut milk (FCM), boiled coconut milk (BCM), and coconut oil. Briefly, the proteins separated using SDS-PAGE were transferred to a nitrocellulose membrane using a mini protein tetra system (Bio-Rad) for 150 min at 60 V constant voltage. The membrane was blocked with phosphate-buffered saline (PBS) containing 0.05% Tween 20 (PBST) containing 5% nonfat milk at 4 °C for 1 h after washing the membrane with PBST. The membrane was reacted with 1:20 dilution of the patient's serum in antibody diluting buffer (5% nonfat milk in PBST) at 4 °C for overnight incubation, after washing with PBST for 5 min three times. The washing step was repeated with PBS for 5 min thrice. The membrane was then reacted at 4 °C for 2 h with the 1:1000 dilution of peroxidase-labeled goat anti-human IgE antibody (Sigma-Aldrich). After washing with PBST for 5 min three times, the membrane was visualized using 4-chloro-napthol substrate.

### Measurement of IgE using Phadia ImmunoCAP test

IgE reactivity to coconut milk was evaluated using Phadia ImmunoCAP (f36) (Thermo Fisher Scientific, Uppsala, Sweden) using the Phadia 100 (Table 1). The test was performed according to the manufacturer’s instructions. The cutoff value was set as > 0.35 kUA/L for sIgE positivity.

### Statistical analysis

Statistical analysis was performed using GraphPad Prism 8.4.3. Percentage of patients’ sera IgE reactivity for each allergen identified in FCM, BCM, WPCO, and DPCO was calculated and analyzed. Patients’ reactivity (%) to allergens identified in each extract was calculated and their median values were compared between patients with anaphylaxis and allergy by the Mann–Whitney *U* test. The median values of total sIgE (kUA/L) of patients with anaphylaxis and allergy were also compared by the Mann–Whitney *U* test. Finally, the correlation (r) between overall reactivity to allergens (%) and total sIgE (kUA/L) of patients with anaphylaxis and allergy was determined by Spearman’s correlation. p-value less than 0.05 (p < 0.05) was considered as statistical significance.

## Results

### Evaluation of the clinical features of patients

Of the patients with coconut milk allergy (18), 10 (56%) were between 1 to 5 years of age. Five (28%) of the patients were under the age of a year. The remaining patients were older than 5 years, with only 1 (6%) patient being older than 18 years. Eleven (61%) of the patients were female, while 7 (39%) were male (Table 1).

Patients with or without anaphylaxis to coconut milk were evaluated according to their cutaneous, respiratory, cardiovascular, and gastrointestinal symptoms. All patients with anaphylaxis (5/18) were female and had urticaria, angioedema, and shortness of breath. Four patients with anaphylaxis had vomited and 1 patient had a cardiac arrest and loss of consciousness. Of the patients without anaphylaxis (13) 11 patients had urticaria and 9 had angioedema. Except for one patient with cough and dysphonia, none of the patients without anaphylaxis had any respiratory symptoms. Furthermore, none of the patients who did not have anaphylaxis experienced any gastrointestinal or cardiovascular symptoms. Apart from coconut milk, one patient experienced red eyes in response to coconut oil fumes, in addition to urticaria and angioedema following the ingestion of coconut oil (Table 1). Seven patients in our study were allergic to other foods. Four patients were allergic to legumes (lentils = 3, soy (in addition to lentils) = 1, chickpea, and pea = 1) and one was allergic to a tree nut (cashew). Three patients were allergic to cow's milk. There was one case each of allergy to house dust mite and hen’s egg (Table 1).

### Protein quantity of the coconut extracts

FCM extract showed the highest concentration of proteins with 41 mg/ml. BCM extract had 24 mg/ml of proteins. Protein concentrations extracted from WPCO and DPCO were 16 mg/ml and 24 mg/ml respectively.

### Protein profiles of the coconut extracts

**Fig. 1:**
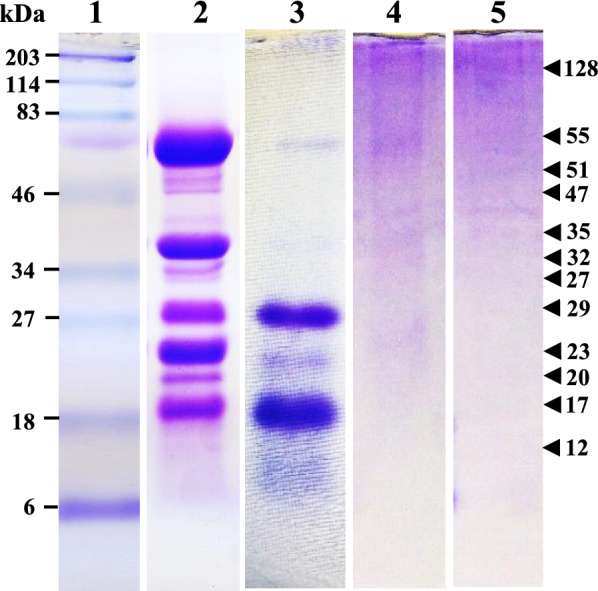
Coomassie-stained SDS-PAGE of coconut extracts; Lane 1: Pre-stained protein markers, Lane 2: FCM, Lane 3: BCM, Lane 4: WPCO, Lane 5: DPCO

In SDS PAGE, 12 and 5 protein bands were detected in FCM and BCM respectively, whereas 4 faint protein bands were identified in WPCO and DPCO (Fig. 1). FCM showed 6 prominent protein bands with molecular weights of 12, 17, 20, 23, 32, and 55 kDa and 6 faint protein bands of 27, 29, 35, 47, 51, and 128 kDa (Fig. 1). BCM had 3 prominent protein bands of 12, 17, and 23 kDa, and 2 faint protein bands of 32 and 55 kDa. Both WPCO and DPCP contained protein bands of 12, 23, 32, and 55 kDa (Fig. 1). Protein bands with molecular weights of 12, 23, 32, and 55 kDa were shared by all four coconut extracts (Fig. 1).

### Allergen profiles of the coconut extracts

**Fig. 2:**
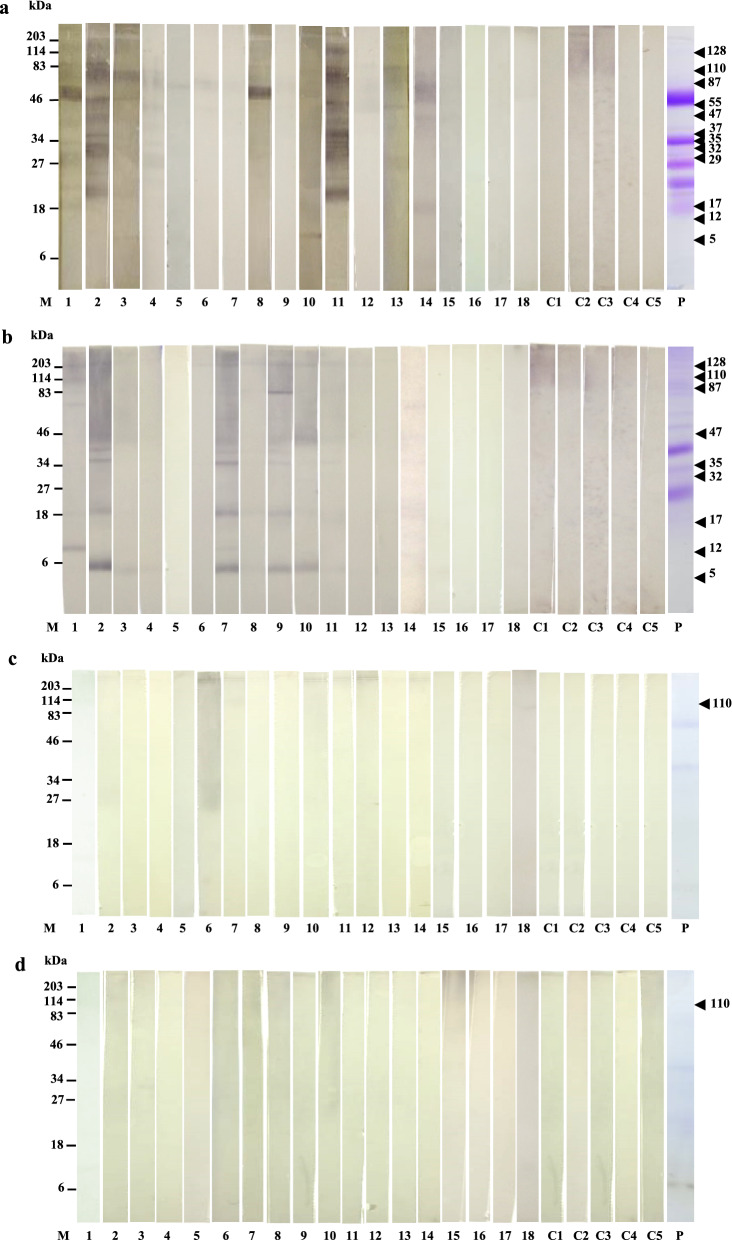
Immunoblotting of coconut extracts; Immunoblots of FCM (**a**) BCM (**b**) WPCO (**c**) and DPCO (**d**); M: Pre-stained protein markers, P: Coomassie stained protein bands, Patients-Anaphylaxis: 1–5, Allergy: 6–18, Healthy controls: C1–C5, All patients’ sera reacted with FCM and BCM, patients 1, 4, 7, 18 reacted with WPCO and only patient no-1 reacted with DPCO

A total of 12 allergens with molecular weights of 5, 12, 17, 29, 32, 35, 37, 47, 55, 87, 110, and 128 kDa were identified in FCM (Fig. 2). In BCM, a total of 9 allergens of 5, 12, 17, 32, 35, 47, 87, 110, and 128 kDa were identified as summarized in Fig. 2. Of all the allergens identified, 5, 12, 17, 32, 35, 47, 87, 110, and 128 kDa allergens were common for both FCM and BCM. However, patients' profiles of immunoreactive bands for FCM and BCM were different (Fig. 2).

**Fig. 3:**
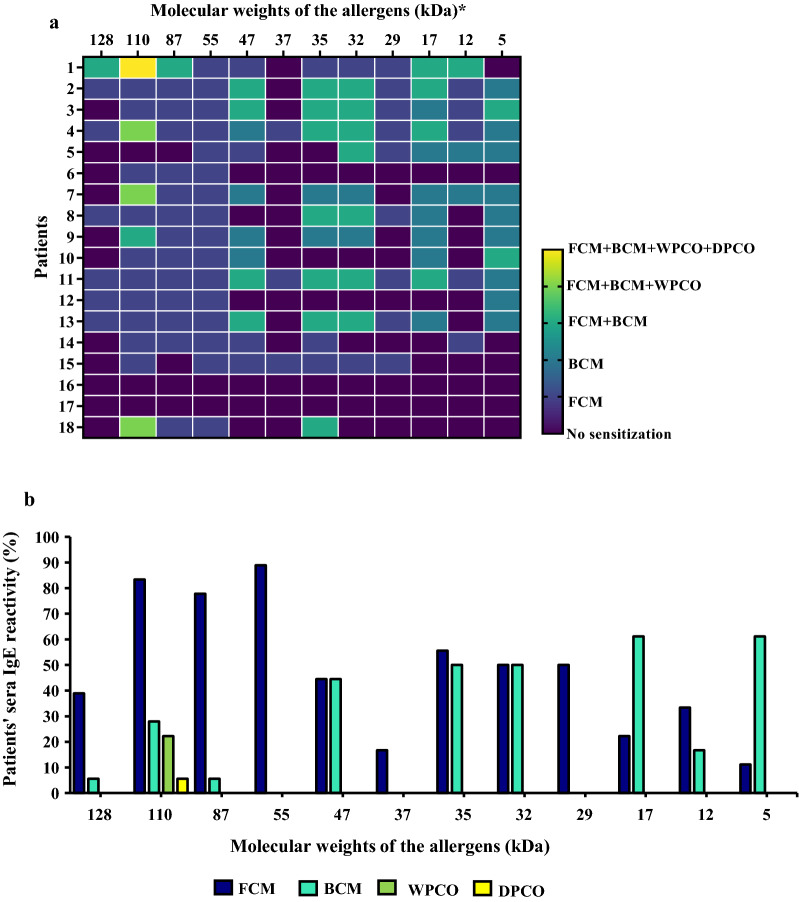
Allergens identified by immunoblotting; Heat map representing allergens and shared allergens identified in FCM, BCM, WPCO and DPCO (**a**), Patient’s sera with IgE reactivity (%) for each allergen identified in FCM, BCM, WPCO and DPCO (**b**)

Interestingly, the majority of patients reacted to 47, 35, 32, 17, 12, and 5 kDa allergens in BCM but not in FCM, whereas the remaining patients recognized the allergens in both FCM and BCM or only in FCM (Fig. 3a).

Only one allergen with 110 kDa molecular weight was identified in WPCO and DPCO which was also present in FCM and BCM (Fig. 3a). Therefore, 110 kDa allergens were identified as the only allergen shared by all four extracts. Four patients reacted to 110 kDa allergen in WPCO (Fig. 3a). Only one patient reacted to the 110 kDa allergen identified in DPCO (Fig. 3a).

Only 1 of 5 patients with anaphylaxis reacted to all four coconut extracts (Fig. 3a). The patient with the highest sIgE level in serum (No. 13) only reacted to half of the total allergens identified (Fig. 3a). The two patients with the lowest sIgE counts (Nos. 16 and 17) did not react to any of the protein extracts (Fig. 3a). With 5 healthy controls, no IgE reactive bands were observed (Fig. 2).

### IgE reactivity (%) of allergens in FCM, BCM, WPCO, and DPCO

In FCM, an allergen of 55 kDa showed the highest IgE reactivity (Fig. 3b). The second highest IgE reactivity was shown by allergens of 110 kDa and 87 kD (Fig. 3b). More than 50% of the patients reacted to the 35 kDa allergen, whereas half of the patients reacted to 29 kDa and 32 kDa allergens. The rest of the allergens identified in FCM showed less than 50% IgE reactivity (IgE reactivity—47 > 128 > 12 > 17 > 37 > 5 kDa) (Fig. 3b).

In BCM, allergens of 17 and 5 kDa showed the highest IgE reactivity. Half of the patients reacted to the 35 kDa and 32 kDa proteins, whereas eight patients reacted to the 47 kDa protein, bringing IgE reactivity closer to 50% (Fig. 3b). A substantial IgE reactivity (less than 50%) was seen with the rest of the allergens identified in BCM (110 > 12 > 128 = 87 kDa) (Fig. 3b).

IgE reactivity of 110 kDa allergen found in WPCO (22%) was higher than in DPCO (6%) (Fig. 3b).

FCM had the highest IgE reactivity of the four coconut extracts tested, while DPCO had the lowest (Fig. 3b). Surprisingly, the percentages of IgE reactivity differed across allergens shared by FCM, BCM, WPCO, and DPCO (Fig. 3b). This contradiction was clearly visible between FCM and BCM. In FCM, most allergens with higher molecular weights (> 50 kDa) had higher IgE reactivity than allergens with lower molecular weights (< 50 kDa), whereas, in BCM, allergens with lower molecular weights (< 50 kDa) had higher IgE reactivity than allergens with higher molecular weights (> 50 kDa) (Fig. 3b).

### Reactivity to allergens (%) by allergic patients with or without anaphylaxis and their total sIgE (kUA/L)

**Fig. 4:**
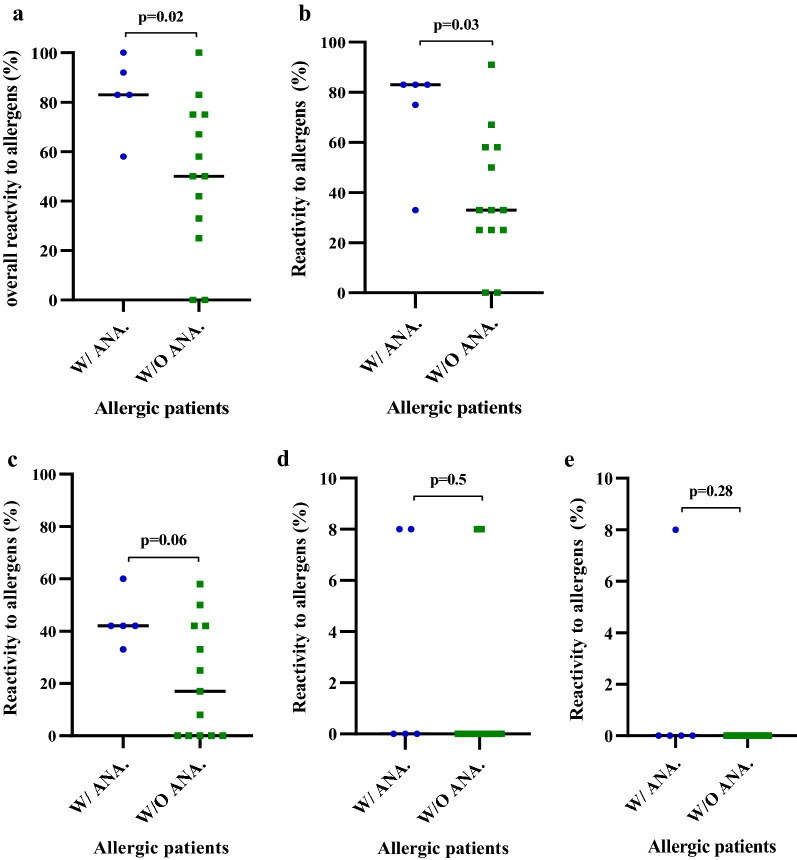
Comparison of reactivity to allergens between allergic patients with anaphylaxis (W/ANA.) and without anaphylaxis (W/O ANA.): Overall percent allergen reactivity (**a**) (p = 0.02), percent reactivity to allergens of FCM (**b**) (p = 0.03), BCM (**c**), WPCO (**d**), DPCO (**e**)

**Fig. 5:**
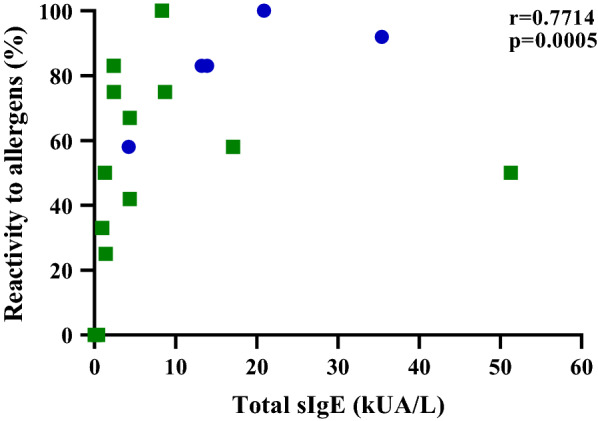
Correlation (r) between total sIgE (kUA/L) and overall reactivity of allergens (%) of allergic patients with anaphylaxis (blue circle) and without anaphylaxis (green square)

There was a clear difference in the medians of reactivity to allergens in FCM between patients with anaphylaxis (83%) and those without (33%) where patients with anaphylaxis showed a higher reactivity (Fig. 4a). Similarly, patients with anaphylaxis (83%) had higher overall reactivity to allergens in all four extracts than patients without anaphylaxis (55%) (Fig. 4b). However, there was no statistically significant difference in medians between patients with and without anaphylaxis in terms of their allergen reactivity in BCM, WPCO, or DPCO (Fig. 4c–e). A statistically significant difference in the medians of total sIgE (kUA/L) to coconut between patients with (14 kUA/L) and without (2 kUA/L) anaphylaxis was observed (Table 1). A positive correlation was observed between overall reactivity to allergens (%) and total sIgE (kUA/L) of total allergic patients with and without anaphylaxis (Fig. 5).

## Discussion

Current knowledge on coconut allergy is limited, as none of the previous studies have determined the allergenicity of BCM, WPCO, and DPCO. This study describes the allergenicity of FCM, BCM, WPCO, and DPCO with 18 patients, 5 with anaphylaxis to coconut milk. The onset of coconut milk allergy in a majority of patients (10/18) was before 5 years and 5/18 developed it in infancy during weaning. All patients developed urticaria, angioedema, or both. Only patients with anaphylaxis experienced shortness of breath, loss of consciousness, cardiac arrest, and vomiting.

In this study, the different protein distributions of FCM, BCM, WPCO, and DPCO were responsible for the unique allergic pattern of each extract. Twelve dietary allergens of coconut milk and oil with 5, 12, 17, 29, 32, 35, 37, 47, 55, 87, 110, and 128 kDa were identified and allergens of 5, 12, 47, 87, 110, and 128 kDa were identified for the first time. FCM and BCM were found to be highly allergenic whereas unrefined WPCO and DPCO had mild allergenicity. The allergens identified would be helpful in the development of Component Resolved Diagnostic (CRD) and therapeutic strategies for coconut such as immunotherapy with modified recombinant proteins.

The proteins identified in SDS-PAGE of FCM (12, 17, 20, 23, 27, 29, 32, 35, 47, 51, 55, and 12 kDa) were similar to previously reported protein distributions from fresh coconut [7, 8, 10, 12, 13, 33, 39–42]. However, the proteins in BCM (12, 17, 23, 32, and 55 kDa), WPCO (12, 23, 32, and 55 kDa) and DPCO (12, 23, 32, and 55 kDa) were observed for the first time.

FCM had all twelve allergens identified while a less number of allergens were detected in BCM (5, 12, 17, 32, 35, 47, 87, 110, and 128 kDa), WPCO (110 kDa), and DPCO (110 kDa). Some of the immunoreactive protein bands identified in immunoblots (5, 37, 87, and 110 kDa) were not visible in SDS-PAGE. This could be due to the fact that these proteins are present in lower concentrations that can only be detected using immunoblotting, a more sensitive method of protein detection than SDS-PAGE. The 55 kDa allergen has been reported previously by Teuber and Peterson [13]. The same study had identified an allergen of 36.5 kDa, which is much closer in terms of molecular weight to the 37 kDa allergen identified in the present study [13]. The 29 kDa allergen found in this study and the 7S storage protein (29 kDa) which has been identified by Benito et al*.* may not be similar allergens [7]. This is because seed storage proteins are heat stable, whereas the 29 kDa allergen which we found was not IgE reactive when coconut milk was boiled. It is possible that allergens with higher molecular weights may split into small fragments while heating. For example, according to Garcia et al. coconut 7S globulin of 156 kDa resolves into 16, 22, and 24 kDa bands on electrophoresis [39]. On the other hand, DeMason, has shown that antibodies raised against 7S globulin in soybean have identified 22 and 67 kDa protein molecules in coconut extract [33]. Therefore, we believe 29 KDa allergen in this study is either a new allergen or a fragment of a larger allergen. Further, molecular studies are needed for elucidation. The 32 and 35 kDa allergens identified in this study possibly belong to 11S globulins (cocasin). As in Carr et al. 11S globulin is a hexamer comprising 54 kDa subunits which makes two distinct bands of 32 and 35 kDa on electrophoresis [26]. DeMason confirmed this by detecting 32 and 35 kDa proteins in coconut using soybean anti 11S globulin antibodies [33]. Further, Garcia et al. have shown in their study that 11S globulin of 326 kDa resolves into two bands of 24 and 34 kDa on electrophoresis [39]. The 17 kDa allergen which we identified in this study may be similar to the 18 kDa allergen which Martin et al*.* and Tella et al*.* have described [9, 11].

Different immunoreactivities were observed in the similar allergens identified in FCM and BCM. This may be due to changes that occur in the structure of proteins while heating which provoke epitope rearrangement. Coconut milk is cooked at different temperatures for different time periods in food preparations. Therefore, people can get sensitized to the same allergen of coconut milk in its different forms (with different molecular structures) which can be a reason for variations in the presence of allergens in FCM and BCM in each patient's allergen profiles. Molecular breakage while boiling was not clearly observed since 9 out of 12 allergens in FCM were observed in BCM. In contrast, 17 kDa allergen along with the novel allergen of 5 kDa showed a higher prevalence in BCM than in the FCM. Hence, these two allergens could have possibly fragmented from a larger allergen while boiling. Furthermore, in patients, allergen-specific IgE is not always formed against the same allergen epitopes. As a result, each patient will react differently to allergens or will not react at all. Despite the differences observed in the allergens of FCM and BCM, this study clearly shows that the allergenicity of coconut milk persists even after boiling, and patients who are allergic to coconut milk should avoid boiled coconut milk.

Except for patient 18, who reacted to 110 kDa in WPCO, none of the patients in this study had a history of coconut oil allergy. However, patients with no history of immediate IgE hypersensitivity reactions to coconut oil had IgE to a 100 kDa in WPCO and DPCO. This may be because higher concentrations of proteins extracted from coconut oil were used in the immunoblots. In Sri Lanka, an average person consumes less than 20 ml of coconut oil per day, which may not contain enough proteins to cause allergy in patients. This study, however, shows that allergy followed by coconut oil ingestion is possible, and patients who are allergic to coconut should be advised to consume refined coconut oil rather than unrefined types. Although WPCO appeared to retain more allergenicity than DPCO in this study, this cannot be stated conclusively because the level of protein or allergen contamination may vary depending on the extraction process. In WPCO extraction, condensed coconut milk is heated for a long time to extract the oil, whereas, in DPCO extraction, coconut is dried in the sunlight until it becomes copra and then pressed against a copra oil expeller. It is difficult to compare which of these extraction processes is more likely to retain protein or allergen contaminants without further research.

Despite the fact that patients with anaphylaxis had a higher reactivity to allergens and total sIgE (kUA/L) than patients without anaphylaxis, this needs to be confirmed with a larger sample size. However, either higher IgE levels or total sIgE cannot be used to determine the severity of allergy. A larger sample size is also required to define any significant relationship between allergen reactivity and total sIgE (kUA/L) in patients with and without anaphylaxis.

Even though coconut is a member of the palm tree family, some of the patients had allergies to legumes and a tree nut. Though coconut allergy is rarely reported among patients with legume and/or tree nut allergy, Teuber and Peterson has shown that clinically relevant cross-reactivity can occur between coconut and walnut (a tree nut) [13]. Similarly, Nguyen et al*.*, has demonstrated that coconut and hazelnut proteins contain cross-reactive allergens [8]. The most likely cause of cross-reactivity between coconut and tree nuts is legumin-like seed storage proteins, which should be investigated further. According to Manso et al*.* and DeMason, both legumin-like seed storage proteins (11 s globulin) and vicilin-like seed storage proteins (7S globulins) are responsible for cross-reactivity between legumes and coconut [10, 33]. A common co-sensitization between coconut and all tree nuts, egg, and wheat, with macadamia nut having the strongest correlation with coconut, has been observed by Kruse et al*.* in their study [3]. However, Stutius et al*.* has shown that children who were sensitized or allergic to peanuts or tree nuts in their study were not more likely to be sensitized or allergic to coconut [4]. According to the authors, cross-sensitivity and clinical cross-reactivity between peanuts and tree nuts with coconut is unlikely [4].

## Conclusions

All the allergens identified in this study have the potential to be used in diagnostic and therapeutic strategies. However, compared to others, the three most predominant allergens with 110 kDa, 87 kDa, and 55 kDa would be the best candidates for CRD. Therefore, these molecules may be used to create a new recombinant coconut ImmunoCAP.

Patients with a history of immediate IgE hypersensitivity to coconut milk should avoid BCM, unrefined WPCO, and DPCO. The amino acid sequences should be determined of all the allergens detected for further categorization.

Further research is needed to investigate the cross-reactivity of tree nuts and legumes with coconut.

## Data Availability

The datasets used and/or analysed during the current study are available from the corresponding author upon reasonable request.

## Abbreviations

4-CN: 4-Chloro naphthol
BCM: Boiled coconut milk
BSA: Bovine serum albumin
CCD: Cross-reactive Carbohydrate Determinants
CRD: Component Resolved Diagnostics
DEA: Diethanolamine
DPCO: Dry processed coconut oil
FCM: Fresh coconut milk
HRP: Hoarse Reddish Peroxidase
IgE: Immunoglobulin E
IUIS: International Union of Immunological Societies
kDa: Kilodalton
kUA/L: Kilo Units of Allergen per Liter
PBS: Phosphate-Buffered Saline
PBST: Phosphate-Buffered Saline, Tween 20
Rf: Retention factor
SDS-PAGE: Sodium Dodecyl Sulphate-Polyacrylamide Gel Electrophoresis
TEA-PGE-3: Triethanolamine-Phenyl glycidyl ether-3
WPCO: Wet processed coconut oil
